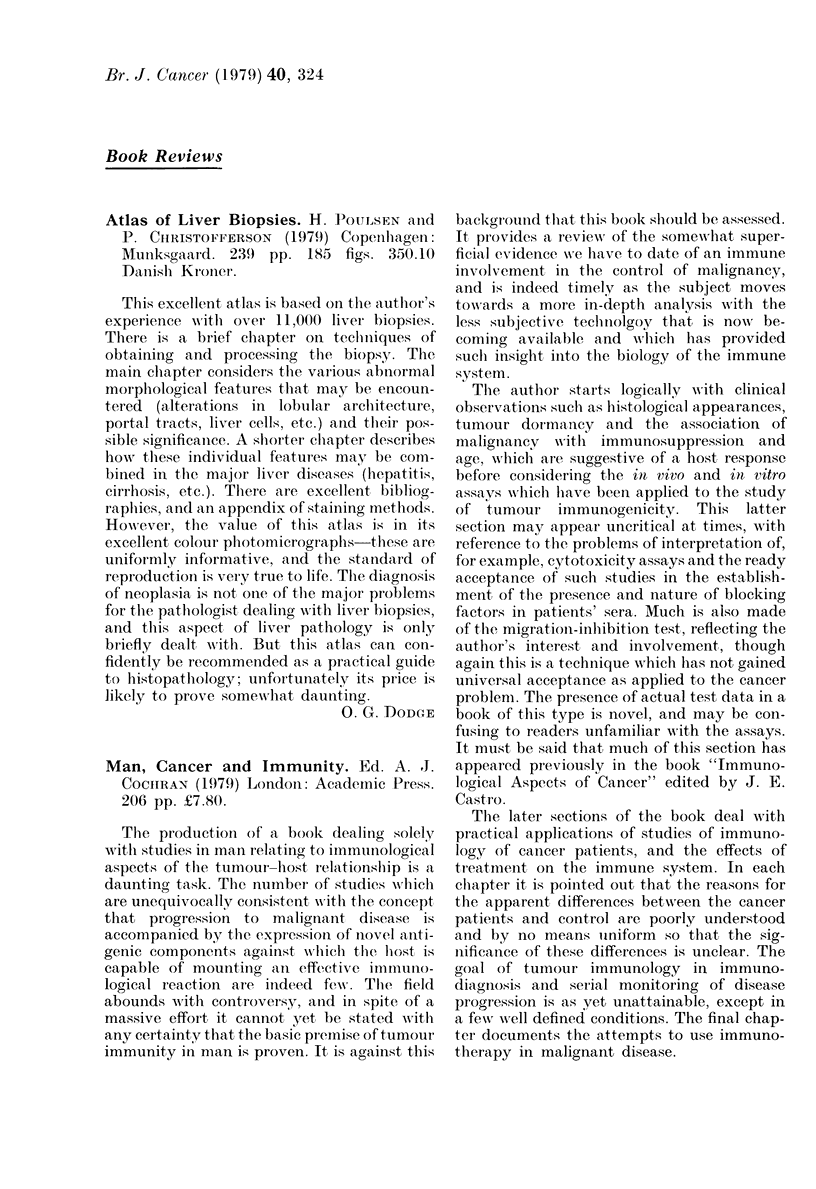# Atlas of Liver Biopsies

**Published:** 1979-08

**Authors:** O. G. Dodge


### *Book Reviews*

**Atlas of Liver Biopsies.** H. POULSEN and P. CHRISTOFFERSON (1979) Copenhagen: Munksgaard. 239 pp. 185 figs. 350.10 Danish Kroner.

This excellent atlas is based on the author's experience with over 11,000 liver biopsies. There is a brief chapter on techniques of obtaining and processing the biopsy. The main chapter considers the various abnormal morphological features that may be encountered (alterations in lobular architecture, portal tracts, liver cells, etc.) and their possible significance. A shorter chapter describes how these individual features may be combined in the major liver diseases (hepatitis, cirrhosis, etc.). There are excellent bibliographies, and an appendix of staining methods. However, the value of this atlas is in its excellent colour photomicrographs—these are uniformly informative, and the standard of reproduction is very true to life. The diagnosis of neoplasia is not one of the major problems for the pathologist dealing with liver biopsies, and this aspect of liver pathology is only briefly dealt with. But this atlas can confidently be recommended as a practical guide to histopathology; unfortunately its price is likely to prove somewhat daunting.

O. G. DODGE